# Elevated CO_2_ affects embryonic development and larval phototaxis in a temperate marine fish

**DOI:** 10.1002/ece3.709

**Published:** 2013-09-04

**Authors:** Elisabet Forsgren, Sam Dupont, Fredrik Jutfelt, Trond Amundsen

**Affiliations:** 1 Norwegian Institute for Nature ResearchP.O. Box 5685 Sluppen, NO-7485, Trondheim, Norway; 2 Department of Biological and Environmental Sciences, University of GothenburgKristineberg 566, SE-45178, Fiskebäckskil, Sweden; 3 Department of Biological and Environmental Sciences, University of GothenburgP.O. Box 463, SE-40530, Göteborg, Sweden; 4 Department of Biology, Norwegian University of Science and TechnologyNO-7491, Trondheim, Norway

**Keywords:** Embryo development, Gobiidae, *Gobiusculus flavescens*, hypercapnia, larval behavior, ocean acidification, two-spotted goby

## Abstract

As an effect of anthropogenic CO_2_ emissions, the chemistry of the world's oceans is changing. Understanding how this will affect marine organisms and ecosystems are critical in predicting the impacts of this ongoing ocean acidification. Work on coral reef fishes has revealed dramatic effects of elevated oceanic CO_2_ on sensory responses and behavior. Such effects may be widespread but have almost exclusively been tested on tropical reef fishes. Here we test the effects elevated CO_2_ has on the reproduction and early life history stages of a temperate coastal goby with paternal care by allowing goby pairs to reproduce naturally in an aquarium with either elevated (ca 1400 μatm) CO_2_ or control seawater (ca 370 μatm CO_2_). Elevated CO_2_ did not affect the occurrence of spawning nor clutch size, but increased embryonic abnormalities and egg loss. Moreover, we found that elevated CO_2_ significantly affected the phototactic response of newly hatched larvae. Phototaxis is a vision-related fundamental behavior of many marine fishes, but has never before been tested in the context of ocean acidification. Our findings suggest that ocean acidification affects embryonic development and sensory responses in temperate fishes, with potentially important implications for fish recruitment.

## Introduction

The anthropogenic increase in atmospheric CO_2_ concentration is a major environmental concern. One alarming consequence is a rapid change in seawater chemistry and decrease of ocean pH (Solomon et al. 2007; Doney et al. 2009), which could have large impacts on marine ecosystems, and pose a threat to marine life (Kerr 2010; Pelejero et al. 2010). Current evidence suggests a range of biological effects in marine organisms, but also considerable variation in the sensitivity to changes in oceanic CO_2_ between and within major taxa (Doney et al. 2009; Kroeker et al. 2010). The variable responses among species and taxa are suggestive of ecosystem effects, but we are currently far from being able to predict how increased CO_2_ affects marine communities (Kerr 2010). It is pivotal to increase our understanding of how parameters related to the fitness of organisms are affected by the on-going ocean acidification. While it is known that increased CO_2_ has negative consequences for many calcifying organisms (Hoegh-Guldberg et al. 2007), effects on fish have been less clear and until recently little studied (Ishimatsu et al. 2008). Compared to invertebrates, fishes have been hypothesized to be more physiologically tolerant to elevated CO_2_ (Pörtner et al. 2004; Melzner et al. 2009). Despite this, there is now evidence of negative fitness consequences of near-future CO_2_ levels on fishes (Munday et al. 2010). Early life history stages seem especially vulnerable to environmental change, including increased CO_2_ levels (Baumann et al. 2012). However, evidence for direct effects of near-future CO_2_ levels on early developmental stages in fish is mixed (Munday et al. 2009a, 2011; Baumann et al. 2012; Frommel et al. 2012). Interestingly, effects on behaviors, such as homing, boldness, activity, and predator avoidance, have been found in coral reef fishes (Munday et al. 2009b, 2010; Dixson et al. 2010; Ferrari et al. 2011, 2012; Simpson et al. 2011), with a resulting higher mortality (Munday et al. 2010; Ferrari et al. 2011). A recent study on adult sticklebacks suggests that also behavior of temperate fishes can be affected (Jutfelt et al. 2013). These changes in behavior can largely be explained by elevated CO_2_ impairing sensory function (Munday et al. 2009b, 2012; Dixson et al. 2010; Simpson et al. 2011). One suggested underlying mechanism, which has received experimental support, is that CO_2_ interferes with brain neurotransmitter function of the GABA-A receptor, leading to neuronal overactivity (Nilsson et al. 2012). Because the GABA-A receptor is the main inhibitory neurotransmitter receptor in the vertebrate brain, increased oceanic CO_2_ levels may lead to multisensory and behavioral impairment in a wide range of fishes (Nilsson et al. 2012).

The scope of this study was to test effects of elevated CO_2_ on fitness-related parameters of a temperate, coastal fish. Such fishes live in habitats with considerable short-term natural variation in pH, in contrast to the open ocean habitat (Hofmann et al. 2011), and may thus be expected to show some tolerance to varying CO_2_ levels (Melzner et al. 2009). Most CO_2_ experiments on marine organisms to date have focused on a single life history stage (Dupont et al. 2012). Here, we tested effects of elevated CO_2_ on successive life history stages (from mating to early larval performance) in an ecologically important fish species in European coastal environments, the two-spotted goby (*Gobiusculus flavescens*). Early life stages are particularly sensitive to environmental stress in teleosts (Brown and Sadler 1989; Kikkawa et al. 2003). We therefore predicted CO_2_ to negatively affect egg survival or embryo development in our experiment. From the hypothesis that exposure to increased CO_2_ levels have effects on brain function and sensory responses (reviewed in Munday et al. 2012), we predicted an effect on behavior via the visual sensory channel. Specifically, based on Nilsson et al. (2012) finding that high CO_2_ causes neuronal over activity, we hypothesized that elevated CO_2_ would lead to sensory hypersensitivity. We therefore predicted increased positive phototaxis as a response to elevated CO_2_.

## Material and Methods

### Model species

**Figure 1:**
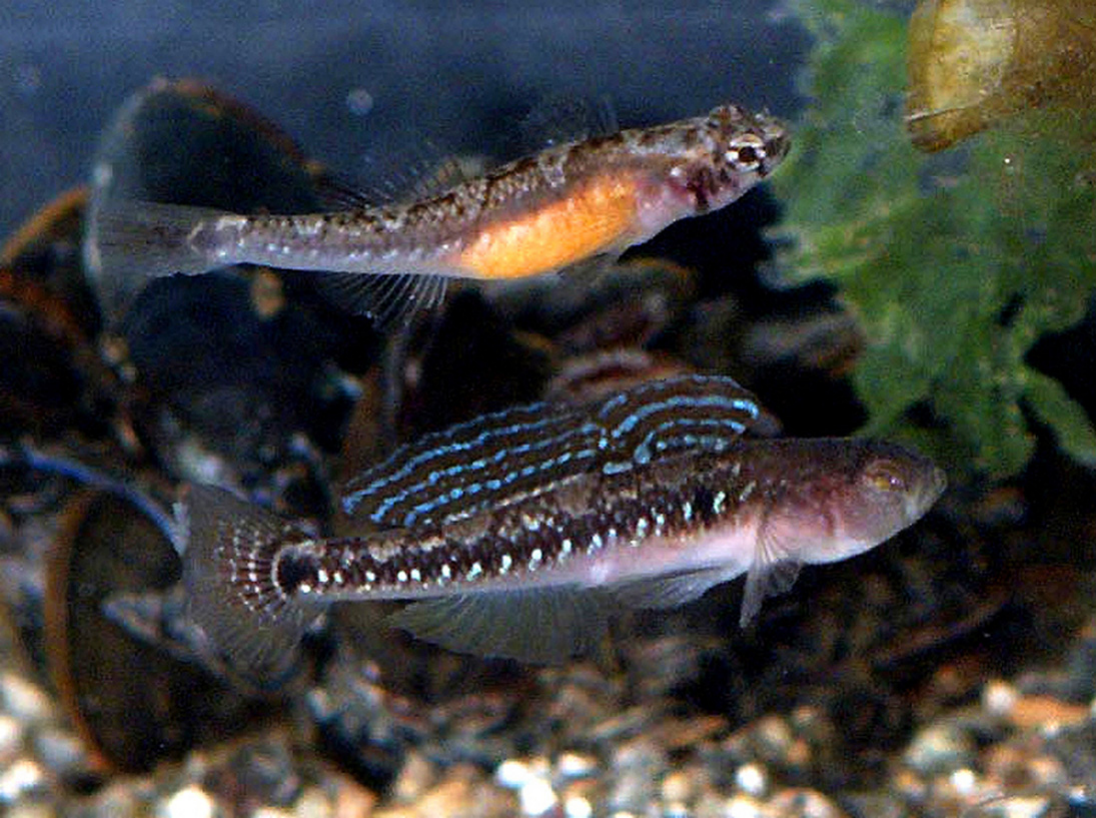
A courting couple of the two-spotted goby, *Gobiusculus flavescens* (female above, male below). Photo by E. Forsgren, from Amundsen and Forsgren (2001), copyright National Academy of Sciences, U.S.A.

The two-spotted goby is a small (ca 5 cm), semi-pelagic, shoaling fish (Fig. 1), belonging to the Gobiidae, one of the largest fish families (ca 2000 species). It is highly abundant along rocky shores of Western Europe, often associated with kelp forests, and is a keystone species in coastal ecosystems of Scandinavia (Salvanes and Nordeide 1993). Two-spotted gobies typically live for 1 year, but can reproduce repeatedly during their single breeding season (May–July). In the breeding season, two-spotted gobies inhabit algal-rich shallow waters (mostly 0–5 m depth) in protected and moderately exposed areas. The reproductive behavior is well known for this species (Amundsen and Forsgren 2001; Forsgren et al. 2004; Myhre et al. 2012). The male takes up a nest site in the kelp vegetation or in an empty mussel shell and attracts females for spawning (Fig. 1). Eggs are laid in a single layer on the substrate, and the male stays to care for (fan, clean, and defend) them until hatching, which takes 1–3 weeks depending on temperature. A male can care for clutches from several females simultaneously in a single nest (Mobley et al. 2009). After hatching, there is no further care and the larvae become pelagic.

The light environment in the shallow algal zone is highly variable with contrasts between darker areas (crevices and shadowed zones behind rocks and kelp) and areas exposed to direct sun light. Light conditions also vary considerably with depth, weather conditions, and time of day. Two-spotted gobies have a single rod visual pigment and three cone pigments, with peak spectral sensitivity at middle wavelengths (Utne-Palm and Bowmaker 2006).

### General experimental procedures

The experiment was carried out at the Sven Lovén Centre for Marine Sciences at Kristineberg, on the west coast of Sweden (58°15′N, 11°27′E), during June and July 2010. Reproductively mature two-spotted gobies were caught using dip nets while snorkeling near the research station, and were brought to the laboratory within 1 h. Male gobies were caught in May and were held together in single-sex storage tanks before being transferred to individual tanks (see below). Females were caught in early June and held together in a storage tank in the laboratory 3–4 days before being introduced to the males (see below). All fish were fed daily with *Artemia* nauplii ad libitum. All replicates (*N* = 28) were run in parallel and had running seawater with a natural variation in temperature between 13 and 16°C (mean 14.9°C). Near the expected hatching time for a clutch the water flow was turned off, causing the temperature to rise to 15–17°C. The aquaria had natural light in addition to artificial light during day time.

On 6 June, all males were introduced to individual aquaria (45 L) with gravel on the bottom, one plastic algae for shelter, and an artificial nest site (a 80 mm long PVC tube, 13 mm diameter, lined with an acetate sheet) tied to a stone. They were allowed 3 days to habituate and establish nest ownership before CO_2_ treatment was initiated (in half of the aquaria; the other half were kept as controls, assigned randomly) and females introduced.

### CO_2_ manipulation

We allowed natural spawning and paternal care to take place in individual aquaria assigned to control (current day ambient pH 8.1; *p*CO_2_ ca 370 μatm; *N* = 14) or elevated CO_2_ (pH ca 7.6; *p*CO_2_ ca 1400 μatm; *N* = 13) treatments. The CO_2_ treatment reflects conditions that may occur during the next century, according to ocean acidification scenarios (Solomon et al. 2007). The elevated CO_2_ treatment was achieved by bubbling CO_2_ into each aquarium (fine bubbles via a wooden air “stone”). Each elevated CO_2_ aquarium had a computerized feedback system (Aqua Medic, CO, http://www.aqua-medic.com) that regulated pH (NBS scale) by addition of pure gaseous CO_2_ directly into the seawater (±0.02 pH units). pH total scale (pH_TS_) and total alkalinity (A_T_) were measured twice a week in each aquarium. Intra-aquarium variability was also assessed by continuous pH_TS_ 12 h measurements once for each aquarium. pH_TS_ was measured with a Metrohm (827 pH lab, Herisau, Switzerland) pH electrode calibrated with salinity adjusted seawater TRIS and AMP buffers, following Dickson et al. (2007). A_T_ measurements were conducted as described by Sarazin et al. (1999) with an accuracy of 10 μmol kg^−1^ seawater. Saturation states (calcite and aragonite) and *p*CO_2_ were calculated from pH_T_ and A_T_ using CO2SYS (Lewis and Wallace 1998) with dissociation constants from Mehrbach et al. (1973) refitted by Dickson and Millero (1987).

A high flow was maintained in each aquarium, minimizing the impact of the experimental system on salinity and A_T_, which was measured in the incoming seawater (average values: 32‰ and 2.32 ± 0.02 mmol/L, respectively). Information on seawater chemistry in each experimental aquarium is provided in Table S1. Generally, treatments were highly consistent in pH, at the levels intended for the experiment. However, two aquaria of the elevated CO_2_ treatment had somewhat lower pH than the rest (7.4 instead of 7.6), but the results for these aquaria did not in any way stand out from the other elevated CO_2_ replicates. For unknown reasons, one replicate failed and had a pH intermediate between the control and the elevated CO_2_ treatment and was therefore excluded from further analyses (reducing sample size to 13 in the elevated CO_2_ treatment). The average pH_TS_ difference between treatments (0.5 units) corresponded to a ca 3.5 times increase in *p*CO_2_.

### Spawning and embryo development

The day after the start of the CO_2_ treatment, a gravid (spawning-ready) female was introduced to each male. Aquaria were inspected several times per day (morning to evening) to record spawning. When spawning was observed or eggs detected, this was noted as the “spawning time”. If spawning did not happen within the first day, a second spawning-ready female was introduced to the male. The female(s) were removed after spawning, and the male left in the aquarium to care for the eggs. Two males, both from the elevated CO_2_ treatment, did not mate. All other males mated and successfully reared clutches to hatching. Most males mated with a single female (*N* = 20) but some (*N* = 5) mated with two females (no difference between treatments). The time until spawning varied from a few hours and up to 2.5 days, except for one case for which 8.5 days elapsed before spawning.

After 9 days post fertilization, and 2 days before expected hatching, clutches were inspected at least twice a day to record hatching. During this period the water flow was turned off, in order to prevent hatched larvae from being washed out of the aquaria. The time when ≥50% of the clutch had hatched was recorded as “hatching time”. As observations of spawning and hatching were made at somewhat irregular intervals over the course of the study, spawning and hatching times were analyzed to the accuracy of 0.5 day, depending on whether a spawning or hatching event was detected before or after 1200 h on a given day. The incubation time was calculated as the time elapsed between spawning and hatching, with a resolution of 0.5 days. The clutch that was initiated ca 8 days after the other clutches was excluded from analyses of incubation time as this clutch experienced somewhat higher water temperatures than the other clutches.

#### Clutch size

Each nest was lined inside with a removable acetate sheet. Hence, spawning females would attach their eggs to the acetate sheet, which could be temporarily removed for photographic documentation of egg numbers (clutch size) and egg/embryo development at various stages between spawning and hatching. Caring males seem in general little affected by such short time disturbance/removal of the clutch, and readily resume care when the clutch is put back. Shortly (0–2 days) after spawning, the acetate sheet with the clutch was carefully removed from the nest, placed in a Petri dish with sea water, and photographed. Thereafter, it was gently put back into the nest again. On day 7 of incubation, we recorded a macro image for assessment of embryonic development of a subset of the embryos. On day 9, we recorded another full-clutch image, in order to calculate clutch size near hatching time and egg loss during incubation. Such egg loss is typically the result of the male removing dead eggs or otherwise eating eggs (filial cannibalism) (Manica 2002; Bjelvenmark and Forsgren 2003). Clutch size “at hatching” in substrate-brooders is usually recorded some time prior to expected hatching, in order not to induce premature hatching.

Manual counting of all eggs/embryos in the images from day 0–2 and day 9 was performed using the count tool in Photoshop CS3 or CS5. On day 7, all abnormal embryos were examined at high magnification. Abnormalities were mainly caused by arrested development at different embryonic stages (i.e., not necessarily dead but not developing properly), and were visually assigned to one of the following categories: unfertilized or arrested at 0–1 days past fertilization (dpf), 2–3 dpf, 4–5 dpf, and 6–8 dpf. In addition, inverted embryos (eyes facing down) were found in a few clutches. The incidence (in %) of each abnormality, as well as total abnormality incidence, within each clutch was then calculated and log_10_ transformed before testing. The proportion of unfertilized eggs on day 9 was very low (only three clutches had >1%, and all had <5% unfertilized eggs).

### Phototaxis

**Figure 2:**
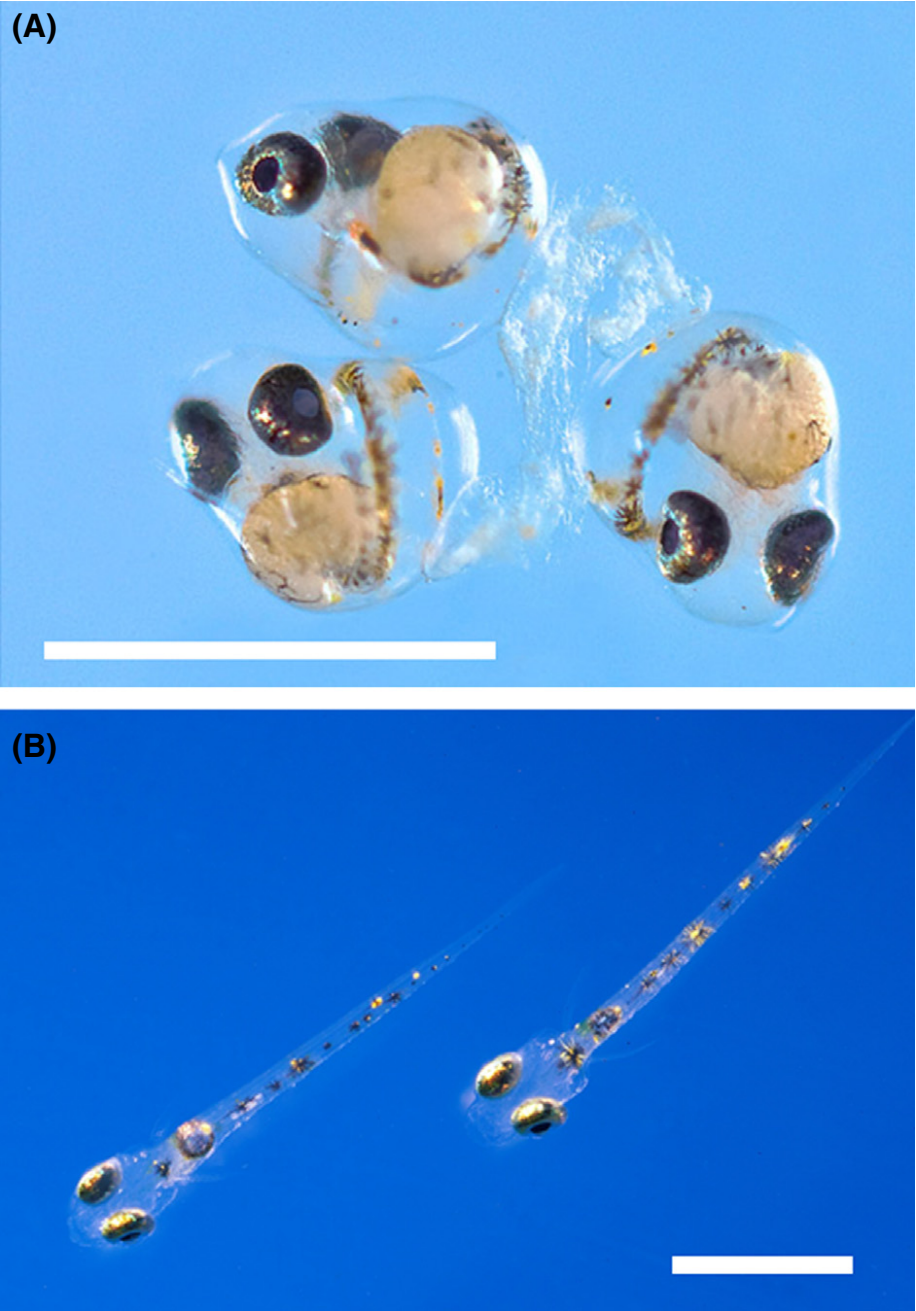
Early life stages of the two-spotted goby, *Gobiusculus flavescens*. (A) Normally developing embryos on day 9 after fertilization. (B) Newly hatched larvae. Scale bars are 1 mm.

In newly hatched larvae (Fig. 2), we tested for effects on positive phototaxis (swimming toward a light source). Phototaxis is a vital behavior of many marine fishes, and is coupled to first feeding and survival (Kamler 1992; Karlsen and Mangor-Jensen 2001). The period between hatching and first feeding in fish larvae is often the most sensitive stage in terms of survival (Cushing 1990; Kamler 1992) and hence an important episode of selection. Larvae of two-spotted gobies hatch with fully pigmented eyes and very small yolk sac reserves, leaving a short time window for exogenous feeding to start in order to prevent irreversible starvation (Kamler 1992). Larval phototaxis was tested in a 50 cm long swim channel, with a 4.5 V LED in one end (Svensson 2006). The test was performed on average 3 h 20 min (SD 2 h 34 min, *N* = 25) after detected hatching (no significant difference between treatments). At the start of a test, 10 randomly selected larvae were taken (using a broad plastic pipette) from a clutch (kept in 1 liter containers after hatching). The larvae were placed in a 10 cm long “start compartment” behind a removable divider that blocked them from swimming out of the compartment. The divider also prevented directional light to reach into the compartment. Immediately after the larvae were placed in the start compartment, the overhead light was turned off (leaving the LED the only light source in the room; hence illuminating only the swim channel, Fig. S1). The removable divider was then lifted, and we recorded by observation the proportion of larvae reaching within 5 cm of the LED in 2 min. This test was repeated for three batches of 10 larvae from each clutch (including all clutches from both treatments), and the mean of these three batches was considered the “clutch phototactic response” (% of larvae reaching the light source). In a subset of clutches (*N* = 14) we also measured the time it took for the fastest larva in each batch to reach the light source. Our visual observation protocol did not allow quantification of details of larval behavior, but the observer followed a total of about 1500 larvae closely (by eye) during the tests, providing general information on swimming behavior. Larvae swimming toward the light were characterized by bursts of swimming interrupted by brief periods of settlement at the bottom, and in many cases also some swimming not directed toward the light source. Fast larvae typically showed a more directional swimming toward the light with less stops on the way. Hence, the phototactic response seems to represent an overall swimming activity rather than swimming speed as such.

#### Repeatability

In order to test for repeatability of the phototactic response, we performed a second phototaxis test on a subset of 12 clutches. The protocol used was identical to that of the initial test. The second test was made on average 12 h 23 min (SD 6 h 54 min, *N* = 12) after the first test, implying 17 h 07 min (SD 9 h 01 min, *N* = 12) after detected hatching. If hatching occurred in the morning, the second test was typically made in the evening of the same day; if hatching occurred in the evening, the second test was typically made in the morning of the following day. The phototactic response of tests 1 and 2 were highly correlated (*r* = 0.78, *N* = 11, *P* < 0.01; one extreme outlier removed), confirming that the recorded phototactic response reflected a true characteristic of larvae from any particular clutch. There was a slight but nonsignificant decrease in the response from the first to the second test (initial test: 49 ± 22% of larvae reaching <5 cm from LED, second test: 43 ± 20%; paired *t*-test, *t* = 1.25, df = 11, *P* = 0.24). The initial and subsequent phototaxis tests were made on different batches of larvae from the same clutch, so that the documented repeatability reflects properties of the clutch rather than of individual larvae.

#### Acute effect?

In our general phototaxis protocol, the swim channel was filled with water from the aquarium from which the test larvae originated (i.e., elevated CO_2_ or control). This was done to mimic a natural situation, as embryos and larvae will be exposed to the same CO_2_ conditions in a future elevated CO_2_ ocean, and also to prevent a sudden (and unnatural) dramatic change of chemical environment for the test larvae. Such a sudden change might have confounded effects of rearing conditions. However, we performed a smaller scale test for any acute response to elevated CO_2_. This was done by testing 24 batches of 10 larvae from a single control clutch, letting 12 batches swim in control and the other 12 in elevated CO_2_ water.

#### General activity

Because previous studies have found increased CO_2_ to lead to increased activity in coral reef fish larvae (Munday et al. 2010), we tested for general levels of activity (unrelated to phototaxis) in uniform overhead light, and in complete darkness. Immediately after the phototaxis test of the third batch of 10 larvae for a given clutch, the overhead light was turned on and the larvae pipetted back into the “start compartment” for activity tests. An overhead light bulb approximately the length of the swim channel ensured uniform and nondirectional light. The divider was lifted and the 10 larvae left for 4 min. We then recorded the proportion of larvae that had left the start compartment. Thereafter, we transferred the larvae back into the start compartment to test for any activity in the dark. We did this by turning off all light sources and again left the clutch for 4 min. Then we recorded any movement as in the light activity test.

The first and second (repeatability) phototaxis tests, as well as the activity tests, were made blindly with respect to treatment (i.e., the observer did not know the treatment of larvae tested). The test for acute responses to elevated CO_2_ was, for logistic reasons, not done blindly.

Statistical analyses were performed with SPSS (version 19; IBM, NY).

## Results

### Spawning and embryonic development

**Figure 3:**
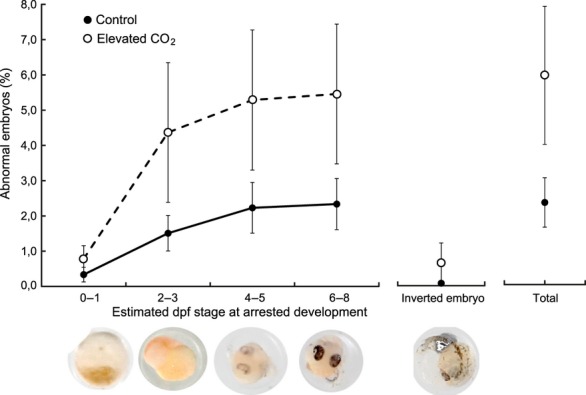
Effect of increased CO_2_ on egg and embryonic development in the two-spotted goby, *Gobiusculus flavescens*. The cumulative proportion (%) of abnormal embryos in developing clutches at day 7 after fertilization in control (*N* = 14) and elevated CO_2_ (*N* = 11) conditions. Values are mean ± 1 SE.

Initial clutch size (only single female spawnings included; mean ± SD) did not differ between treatments (control: 1392 ± 302 eggs, CO_2_: 1434 ± 255 eggs; *t* = 0.33, df = 18, *P* = 0.42). Egg incubation time varied from 9.5 to 12.5 days, but with no detectable effect of treatment (control: 11.2 ± 0.9 days, CO_2_: 11.4 ± 0.7 days; *t* = 0.39, df = 22, *P* = 0.70). Clutch size at day 9, was also relatively similar between treatments (control: 1556 ± 600 eggs, CO_2_: 1410 ± 594 eggs; *t* = −0.60, df = 23, *P* = 0.55). The proportion of eggs lost before hatching (i.e., loss of eggs between spawning and day 9) was, however, more than twice as high in the elevated CO_2_ treatment (14.5 ± 13.3%) as in the control (6.4 ± 5.4%; Mann–Whitney *U* test, *P* = 0.025). Even if most eggs and embryos developed normally (Fig. 2), inspection for abnormal embryos at high magnification (day 7) showed several instances of abnormalities, which mainly were caused by arrested development at various embryonic stages (Fig. 3). Abnormal embryonic development occurred more than twice as frequently in the elevated CO_2_ (6.1 ± 6.7%) as in the control (2.4 ± 2.7%) treatment (*t* = −2.09, df = 23, *P* = 0.048) (Fig. 3).

### Larval phototaxis

A high proportion of the larvae showed a strong directional phototactic response, and swam quickly toward the light (mean time to swim 50 cm: 52 ± 22 sec [*N* = 14]; fastest larva: 13 sec). Calculated in terms of body lengths (mean SL at hatching 2.8 mm; Svensson 2006), the mean speed was 3.4 BL s^−1^, and 13.7 BL s^−1^ for the fastest larvae. Hence, the phototactic response of the larvae was very strong.

**Figure 4:**
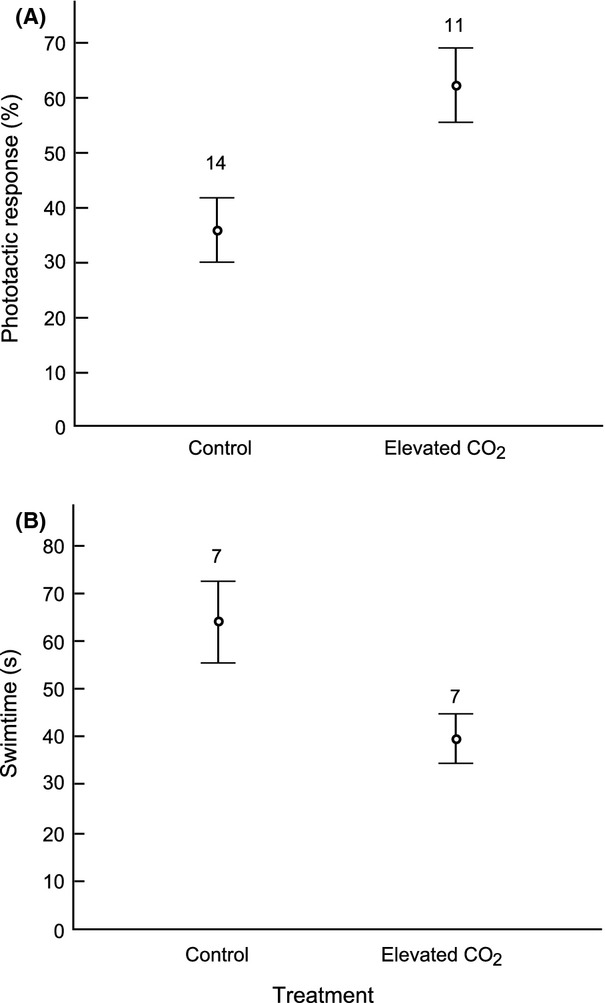
Effect of increased CO_2_ on phototaxis of newly hatched larvae of the two-spotted goby *Gobiusculus flavescens*. (A) phototactic response, shown as percent of larvae reaching a LED light 50 cm away in 2 min, and (B) the time (s) used by the fastest larva in each batch of 10 to reach the light. The larvae had developed from fertilization to hatching in control or elevated *p*CO_2_ treatments, respectively. Values are mean ± 1 SE.

Larvae reared under elevated CO_2_ conditions showed a much stronger phototactic response than those reared under control conditions. In the test apparatus, 62 ± 22% of larvae from the elevated CO_2_ treatment reached the light source within 2 min, as compared to only 36 ± 22% of the control larvae (*t* = 2.99, df = 23, *P* = 0.007) (Fig. 4). The time used by the fastest larva of each clutch to reach the light also revealed a marked difference between treatments. On average, the fastest larvae from the elevated CO_2_ clutches used 40 ± 14 sec to reach the light, compared to 64 ± 22 sec for control treatment larvae (*t* = −2.47, df = 12, *P* = 0.029) (Fig. 4).

#### Acute effect?

There was no evidence of an acute effect of *p*CO_2_, as neither the overall phototactic response (percent reaching the light source; elevated CO_2_: 35 ± 14%, control 41 ± 23%, *t* = −0.75, df = 22, *P* = 0.46) nor the swimming speed of the fastest larva (elevated CO_2_: 69 ± 23 sec, control 71 ± 26 sec, *t* = 0.14, df = 22, *P* = 0.89) differed between elevated CO_2_ and control test environments. For both test environments, the levels were as expected for a clutch that had developed in control CO_2_ conditions (see Fig. 4). Hence, current test conditions (*p*CO_2_ in the swim channel) did not affect the results.

#### General activity

Under uniform overhead light, activity was low and not different between rearing treatments (ca 20% leaving the start compartment, *P* > 0.8 between treatments). Activity in the dark was very low. In only one case did a larva move into the neighboring chamber (0.4% of larvae). Hence, the larvae showed minimal activity in the dark and also very limited directional swimming in the light when not exposed to a directional light source.

## Discussion

Our results revealed strong negative effects of elevated CO_2_ on embryonic development and egg loss. There were no apparent effects on the more conventional measures of spawning and clutch sizes, though, our exposure time to the treatments were very short before spawning. Unlike most other studies of CO_2_ effects on wild animals, our study established an environment where natural reproductive behaviors could take place (spawning and embryo development during parental care), adding to the ecological relevance of the results. While embryonic malformations were likely a direct effect of elevated CO_2_, the cause of egg loss may have multiple causes. Possible explanations include that the parental male removed (ate) embryos not developing normally, or that elevated CO_2_ resulted in aberrant parental care behavior. Our results are in contrast to some studies on fishes reporting no negative effects of elevated CO_2_ on egg development (Munday et al. 2009a; Franke and Clemmesen 2011), but in line with recent findings for the temperate Atlantic fish *Menidia beryllina* for which elevated CO_2_ caused considerable mortality on the egg stage and a higher percentage of malformations in newly hatched larvae (Baumann et al. 2012). This suggests that early life stages can be negatively impacted by elevated CO_2_, but also that effects may vary among species. Marine fish larvae face a very high mortality, and mortality rates during larval and early juvenile stages significantly affect recruitment (Cushing 1990; Houde 1997). However, the potential effects of elevated CO_2_ on the population level remain to be investigated.

Experimentally elevated CO_2_ conditions caused a substantially stronger phototactic response in newly hatched larvae. This was not a general activity effect, but a specific effect on the response to a light stimulus, as larvae exposed to uniform overhead illumination showed a low level activity, which was not different between rearing treatments. In the dark almost no activity was detected. Moreover, we did not find any support for an acute effect of CO_2_ concentration of the test water. Thus, phototaxis was affected by CO_2_ conditions during embryonic development.

Positive phototaxis is generally considered to confer fitness benefits to fish larvae (Kamler 1992; Karlsen and Mangor-Jensen 2001). However, the effect of increased CO_2_ on the phototactic response in our study was very strong, with a 65–70% increase in two different measures. Such a strong response may suggest visual hypersensitivity caused by elevated CO_2_, in line with the idea that high CO_2_ causes neuronal overactivity due to effects on brain neurotransmitter function (Nilsson et al. 2012). Visual sensitivity in animals is likely under stabilizing selection, with low sensitivity leading to an absence of an appropriate response, and hypersensitivity leading to maladaptive responses to weak or absent signals (Bradbury and Vehrencamp 2011). This is the first time a CO_2_ effect on phototaxis is tested. Further investigations are needed to establish whether the dramatic increase in phototactic response has positive or negative fitness effects for the larvae, and what the potential implications on the population level are. However, it is not unlikely that visual hypersensitivity is costly, as hypersensitive larvae may maladaptively respond to the many weak and accidental light stimuli in the complex visual environment of rocky shores kelp forests. Given that swimming toward the light was not monotonous, but interrupted by more or less frequent stops and turns by the larvae, we suggest that the increased phototactic response was likely caused by an increased sensory drive and a more directional and continuous swimming.

Larval phototaxis was affected by CO_2_ conditions during embryonic development but not by whether the test was conducted in elevated CO_2_ versus control water. This is consistent with previous work suggesting that short-term elevations of CO_2_ do not cause behavioral effects in coral reef fish larvae (Munday et al. 2010; Nilsson et al. 2012). Together with the present study, these works suggest that chronically elevated CO_2_ concentrations in a future ocean acidification scenario can be much more serious for fish than the sometimes large but short-term natural fluctuations that fish in many habitats (e.g., temperate coastal) experience today (Hofmann et al. 2011). When it comes to long-term effects of elevated CO_2_, a crucial question is whether organisms over multiple generations may adapt by selection of more tolerant genotypes and/or acclimate to climate stressors. It remains to be seen whether organisms with relatively long generation times, like fishes, have the potential to evolutionarily adapt to the rapidly increasing ambient CO_2_ levels. Results from tropical reef fish suggest that nongenetic parental effects may allow some acclimation to elevated CO_2_ over generations (Miller et al. 2012), but the long-term potential and generality of any such effect remains to be investigated.

In summary, the study reveals clear effects of elevated CO_2_ on embryos and larvae of a temperate goby. Increased CO_2_ resulted in higher egg loss and a higher incidence of abnormalities among developing embryos, in line with the prediction that negative effects should be evident at early life stages. We identified effects of elevated CO_2_ on vision-related behavior (phototactic response), as predicted by the hypothesis that CO_2_ affects sensory responses generally (see Munday et al. 2012). Specifically, newly hatched larvae reared under elevated CO_2_ conditions showed a dramatically increased phototactic response, in line with the recent finding by Nilsson et al. (2012) that high CO_2_ causes neuronal “overactivity”. Together with other recent findings (e.g., Baumann et al. 2012; Munday et al. 2012; Jutfelt et al. 2013), our results suggest that marine fishes are not as tolerant to increasing CO_2_ as previously thought. Notably, the effects of CO_2_ on sensory-related fish behavior seem not to be restricted to tropical reef fishes but may instead be of global concern.

## Supporting Information

**Table S1.** Seawater chemistry parameters (mean ± SE) in individual aquaria of the experiment. In aquaria 16 and 20 no spawning occurred. Aquarium 24 was not included in analyses due to failure of the treatment. All these three aquaria were elevated CO_2_ treatments. Hence, analyses are based on 14 aquaria with control conditions, and 11 elevated *p*CO_2_ aquaria.

**Figure S1.** Swim channel for phototaxis test. The 50 cm swim channel with a LED light in the far end used in the experiment to test phototactic response of newly hatched two-spotted goby larvae. The section to the left represents the “start chamber”, with the blocking divider removed (see text).
