# Supplementary material for: Elevated CO2 affects embryonic development and larval phototaxis in a temperate marine fish
Source: Ecol Evol. 2013 Sep 4;3(11):3637–46. doi: 10.1002/ece3.709 (PMC3810864; doi:10.1002/ece3.709)
Supplement: Supplementary file 1 [file ece30003-3637-SD1.docx]

Supporting information

**Table S1.** Seawater chemistry parameters (mean ± s.e.) in individual aquaria of the CO_2_ experiment on two-spotted gobies (*Gobiusculus flavescens*). In aquaria 16 and 20 no spawning occurred. Aquarium 24 was not included in analyses due to failure of the treatment. All these three aquaria were elevated CO_2_ treatments. Hence, analyses are based on 14 aquaria with control conditions, and 11 elevated pCO_2_ aquaria.

|  |  |  | Calculated | | | | |
| --- | --- | --- | --- | --- | --- | --- | --- |
| Aquarium | Treatment | pH_TS_ | pCO_2_  μatm | [HCO3^-^]  μM.Kg^-1^ | [CO3^--^]  μM.Kg^-1^ | Ωcalcite | Ωaragon |
| average | Control | 8.09±0.03 | 367±2 | 1902±1 | 162.1±0.6 | 3.94±0.01 | 2.52±0.01 |
| average | High CO_2_ | 7.56±0.01 | 1432±28 | 2164±2 | 55.4±0.7 | 1.35±0.02 | 0.86±0.01 |
| 1 | High CO_2_ | 7.61±0.01 | 1268±38 | 2152±4 | 60.3±1.5 | 1.47±0.04 | 0.94±0.02 |
| 2 | Control | 8.10±0.01 | 357±8 | 1895±6 | 165.2±2.5 | 4.01±0.06 | 2.56±0.04 |
| 3 | High CO_2_ | 7.59±0.01 | 1315±19 | 2157±2 | 58.1±0.7 | 1.41±0.02 | 0.90±0.01 |
| 4 | Control | 8.09±0.01 | 373±8 | 1907±6 | 160.2±2.4 | 3.89±0.06 | 2.49±0.04 |
| 5 | High CO_2_ | 7.58±0.01 | 1365±26 | 2161±2 | 56.3±1.0 | 1.37±0.02 | 0.87±0.01 |
| 6 | Control | 8.10±0.01 | 363±7 | 1899±5 | 163.3±2.1 | 4.00±0.05 | 2.53±0.03 |
| 7 | High CO_2_ | 7.42±0.02 | 2012±109 | 2201±5 | 40.4±1.8 | 0.98±0.04 | 0.63±0.03 |
| 8 | Control | 8.08±0.01 | 376±4 | 1909±3 | 159.2±1.3 | 3.87±0.03 | 2.47±0.02 |
| 9 | Control | 8.09±0.01 | 371±7 | 1905±6 | 160.8±2.7 | 3.91±0.06 | 2.50±0.04 |
| 10 | Control | 8.10±0.01 | 358±6 | 1896±5 | 164.8±2.0 | 4.01±0.05 | 2.56±0.03 |
| 11 | High CO_2_ | 7.59±0.01 | 1324±46 | 2157±5 | 58.2±1.8 | 1.41±0.04 | 0.90±0.03 |
| 12 | High CO_2_ | 7.59±0.01 | 1316±41 | 2157±4 | 58.4±1.6 | 1.42±0.04 | 0.91±0.02 |
| 13 | Control | 8.09±0.01 | 366±8 | 1902±6 | 162.4±2.5 | 3.95±0.06 | 2.52±0.04 |
| 14 | Control | 8.09±0.91 | 373±7 | 1907±5 | 160.2±2.2 | 3.89±0.05 | 2.48±0.03 |
| 15 | High CO_2_ | 7.59±0.01 | 1319±39 | 2157±4 | 58.2±1.5 | 1.42±0.04 | 0.90±0.02 |
| 16 | High CO_2_ | 7.59±0.01 | 1315±49 | 2156±5 | 58.6±2.0 | 1.42±0.05 | 0.91±0.03 |
| 17 | Control | 8.09±0.01 | 365±8 | 1901±6 | 162.7±2.5 | 3.95±0.06 | 2.52±0.04 |
| 18 | High CO_2_ | 7.60±0.01 | 1294±38 | 2155±4 | 59.2±1.5 | 1.44±0.04 | 0.92±0.02 |
| 19 | Control | 8.11±0.01 | 354±7 | 1892±5 | 166.1±2.2 | 4.04±0.05 | 2.58±0.03 |
| 20 | High CO_2_ | 7.60±0.01 | 1299±36 | 2155±4 | 59.0±1.4 | 1.43±0.04 | 0.92±0.02 |
| 21 | Control | 8.09±0.01 | 372±7 | 1906±5 | 160.5±2.2 | 3.90±0.05 | 2.49±0.03 |
| 22 | High CO_2_ | 7.58±0.01 | 1375±41 | 2162±4 | 56.1±1.5 | 1.36±0.04 | 0.87±0.02 |
| 23 | Control | 8.09±0.01 | 369±7 | 1904±5 | 161.4±2.1 | 3.92±0.05 | 2.50±0.03 |
| 25 | Control | 8.10±0.01 | 360±8 | 1897±7 | 164.3±2.6 | 3.99±0.06 | 2.55±0.04 |
| 26 | High CO_2_ | 7.42±0.03 | 2063±161 | 2201±6 | 40.3±2.6 | 0.98±0.06 | 0.63±0.04 |
| 27 | Control | 8.08±0.01 | 378±7 | 1911±5 | 158.7±2.1 | 3.87±0.05 | 2.46±0.03 |
| 28 | High CO_2_ | 7.58±0.01 | 1350±56 | 2159±5 | 57.4±2.1 | 1.40±0.05 | 0.89±0.03 |


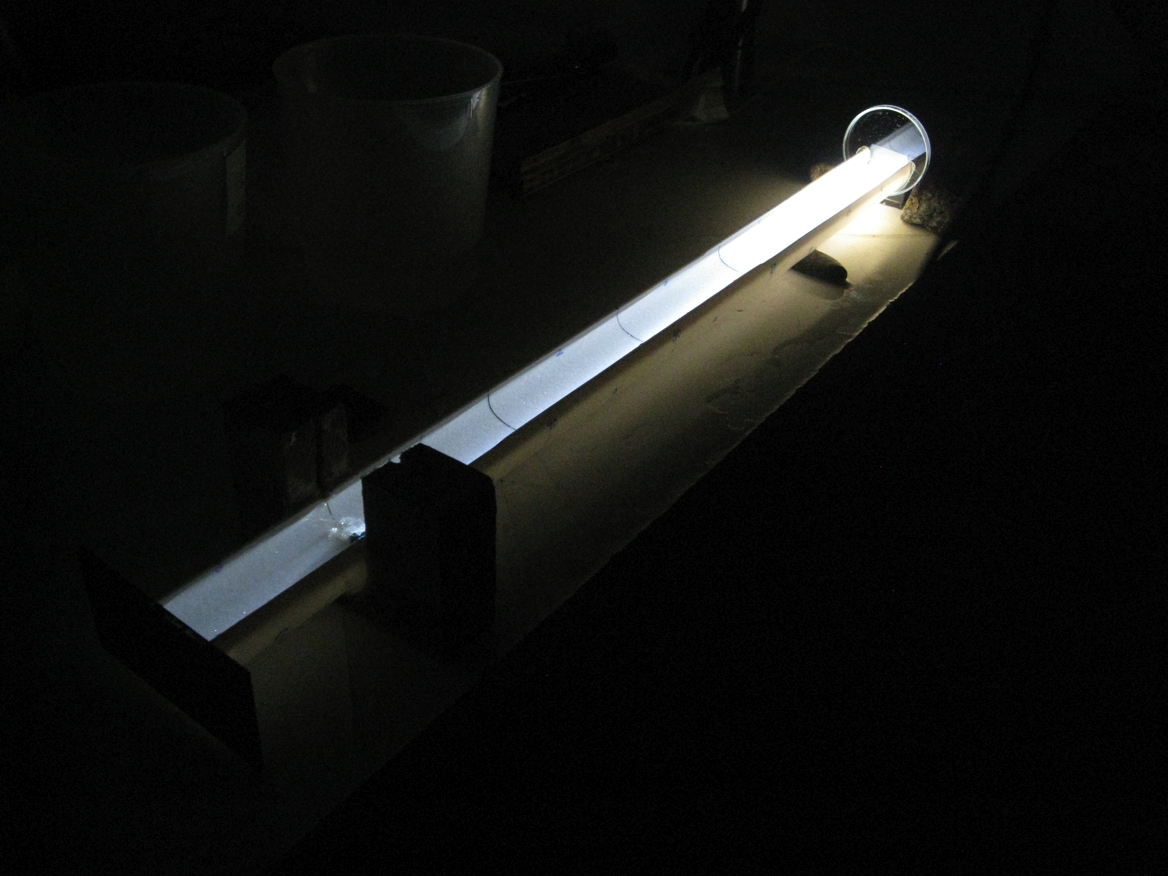


**Figure S1.** Swim channel for phototaxis test. The 50 cm swim channel with a LED light in the far end used in the experiment to test phototactic response of newly hatched two-spotted goby larvae. The section to the left represents the “start chamber”, with the blocking divider removed (see text).
